# A note on competing risks in survival data analysis

**DOI:** 10.1038/sj.bjc.6602102

**Published:** 2004-08-10

**Authors:** J M Satagopan, L Ben-Porat, M Berwick, M Robson, D Kutler, A D Auerbach

**Affiliations:** 1 Department of Epidemiology and Biostatistics, Memorial Sloan-Kettering Cancer Center, New York 10021, USA; 2 Division of Epidemiology, Cancer Research Facility, University of New Mexico, Albuquerque, NM 872131, USA; 3 Clinical Genetics Service, Department of Medicine, Memorial Sloan-Kettering Cancer Center, New York 10021, USA; 4 Department of Otolaryngology, New York University Medical Center, New York 10021, USA; 5 Laboratory of Human Genetics and Hematology, The Rockefeller University, New York 10021, USA

**Keywords:** cumulative incidence, informative censoring, Kaplan–Meier estimate, overall survival probability

## Abstract

Survival analysis encompasses investigation of time to event data. In most clinical studies, estimating the cumulative incidence function (or the probability of experiencing an event by a given time) is of primary interest. When the data consist of patients who experience an event and censored individuals, a nonparametric estimate of the cumulative incidence can be obtained using the Kaplan–Meier method. Under this approach, the censoring mechanism is assumed to be noninformative. In other words, the survival time of an individual (or the time at which a subject experiences an event) is assumed to be independent of a mechanism that would cause the patient to be censored. Often times, a patient may experience an event other than the one of interest which alters the probability of experiencing the event of interest. Such events are known as competing risk events. In this setting, it would often be of interest to calculate the cumulative incidence of a specific event of interest. Any subject who does not experience the event of interest can be treated as censored. However, a patient experiencing a competing risk event is censored in an informative manner. Hence, the Kaplan–Meier estimation procedure may not be directly applicable. The cumulative incidence function for an event of interest must be calculated by appropriately accounting for the presence of competing risk events. In this paper, we illustrate nonparametric estimation of the cumulative incidence function for an event of interest in the presence of competing risk events using two published data sets. We compare the resulting estimates with those obtained using the Kaplan–Meier approach to demonstrate the importance of appropriately estimating the cumulative incidence of an event of interest in the presence of competing risk events.

Survival analysis is the analysis of data measured from a specific time of origin until an event of interest or a specified endpoint (Collett, 1994). For example, in order to determine the incidence of death due to breast cancer among breast cancer patients, every patient will be followed from a baseline date (such as date of diagnosis or date of surgery) until the date of death due to breast cancer or study closing date. A patient who dies of breast cancer during the study period would be considered to have an ‘event’ at their date of death. A patient who is alive at the end of the study would be considered to be ‘censored’. Thus, every patient provides two pieces of information: follow-up time and status (i.e., event or censored). However, a patient can experience an event different from the event of interest. For example, a breast cancer patient may die due to causes unrelated to the disease. Such events are termed competing risk events. Survival data are often summarised using the cumulative incidence function for an event. The goal of this paper is to illustrate to the clinical investigator the nonparametric estimation of the cumulative incidence for an event of interest in the presence of competing risk events.

## GENERAL CONCEPTS IN SURVIVAL ANALYSIS

Clinical studies often focus on estimating the survivor function or the overall survival probability. This is the probability of being event-free at least up to a given time. The event is any specific event of interest. The overall survival probability is estimated using the person follow-up time and event status. The survival at a given time is the conditional probability of surviving to a specific time given that the individual is at risk for the event (such as mortality) at that time. This is estimated as the ratio of the number of individuals that are event-free at that time to the number of individuals that lived event-free at least up to that time. Henceforth, when referring to survival at a given time, we in fact mean survival conditional upon being at risk at that time.

For example, let us consider the event of interest to be death. Suppose 100 breast cancer patients lived for at least 1 year following surgery and five patients died at the end of the first year. The estimated survival at 1 year is 95%. This estimate is the survival probability conditional upon the fact that the 95 surviving patients were all at risk at 1 year. Suppose at 2 years, 10 more patients die. The estimated survival at 2 years is 85/95=89.5%. The estimated overall survival up to and including 2 years is the probability of having survived the first year and the second year. This is calculated as 95^*^89.5=85%. Therefore, the overall survival or the probability of surviving up to 2 years is 85%. This is the basis for the Kaplan–Meier estimate of survival probability (Kaplan and Meier, 1958).

Equivalently, we can consider estimating the incidence of mortality among patients with breast cancer. In the above example, the estimated incidence of mortality at 1 year is five out of 100 patients, that is, 5%. The probability of dying at 2 years is the probability of living past the first year and dying during the second year. Note that 95 of 100 patients survived past the first year, and 10 of these 95 patients died in the second year. Therefore, the estimated incidence of mortality at 2 years is 95/100^*^10/95=10%. The cumulative incidence of mortality up to a given time is the probability that an individual dies by that time. This is the sum of mortality incidences occurring up to that time. Therefore, the cumulative incidence of mortality at 2 years is the sum of mortality incidences in the first and second years, that is, 5+10=15%. This is simply the converse of survival. In other words, the cumulative incidence of an event at a given time is one minus the overall survival probability at that time.

An investigator may be interested in examining outcomes other than mortality, such as incidence of disease recurrence or incidence of a second primary cancer. In this scenario, a patient is followed from a baseline time (such as date of diagnosis or date of treatment) until the outcome of interest. In order to estimate the incidence of ovarian cancer among breast cancer patients, any patient who does not develop ovarian cancer is treated as censored. If a woman has not developed ovarian cancer by the study closing date, she would be considered censored. If a woman dies of breast cancer prior to developing ovarian cancer, she would be considered censored at her date of death when evaluating the incidence of ovarian cancer. However, the latter type of censoring is informative since this patient is censored due to the occurrence of an intervening event (mortality). Such intervening events are known as ‘competing risk events’. Events other than death can also be competing risk events. Suppose a breast cancer patient undergoes a prophylactic oophorectomy after surgery for breast cancer. This prophylactic treatment substantially reduces the probability of developing ovarian cancer, and hence is treated as a competing risk event when calculating ovarian cancer incidence. Thus, a competing risk event may preclude the onset of the event of interest, or may modify the probability of the onset of the event of interest. Other types of data may also arise in practice. For example, a patient may also experience multiple or repeated events such as cancer in a different site (for example, primary melanoma in a breast cancer patient), second primary cancer or disease recurrence. In this paper, we restrict ourselves to competing risk events where the follow-up duration of a patient ends at the onset of the first event, and do not focus on multiple or repeated events occurring in a patient. We first describe the two illustrative examples, and then demonstrate the estimation of cumulative incidence of an event of interest.

## ILLUSTRATIVE EXAMPLES

### Fanconi anaemia (FA) data

A total of 755 patients were identified through the International Fanconi Anaemia Registry at The Rockefeller University (Kutler *et al*, 2003). Complementation group was determined for a subset of these patients by cDNA transduction and/or mutation analysis. The patients were followed from their date of birth (i.e., the date of onset of FA) until the onset of haematologic malignancy (HM) or the last follow-up date (in months). Several patients died due to FA-related complications without developing HM. Hence, death prior to the onset of HM is a competing risk event.

Some of the FA patients developed other malignancies during their follow-up period. In order to simplify the presentation, and to demonstrate the calculations of interest, here we focus on HM as the event of interest, and death prior to the onset of HM as the competing risk event, and do not consider information on other malignancies (i.e., multiple or repeated events).

### Breast cancer data

This data set consists of 305 women diagnosed with breast cancer at any age, self-identified as Ashkenazi Jewish (Robson *et al*, 1999). All these women underwent breast-conservation therapy at MSKCC between January 1980 and December 1990. The tissue samples were examined for the presence of a 185delAG or 5382insC mutation in *BRCA1* and 6174delT mutation in *BRCA2*, the mutations commonly occurring in Ashkenazi Jewish individuals. These women were followed from their date of breast-conservation therapy until death due to breast cancer or the last follow-up date. Several patients died due to causes other than breast cancer. Hence, death due to other causes is a competing risk for breast cancer specific mortality.

## METHODS

In this section, we describe estimating the cumulative incidence function for an event of interest in the presence of competing risk events. Below we first discuss nonparametric estimation using the Kaplan–Meier approach. Patients who are alive at the end of the study as well as patients experiencing competing risk events are all considered censored in the same manner under this estimation approach. The Kaplan–Meier approach is based on the premise that the censoring is noninformative, that is, independent of the event of interest. However, the presence of competing risk events result in informative censoring. We next outline how to nonparametrically estimate the cumulative incidence of the event of interest by differentiating between the informative and noninformative censoring.

### The Kaplan–Meier estimate of cumulative incidence

The Kaplan–Meier approach (Kaplan and Meier, 1958), also known as the product-limit estimate, provides a nonparametric estimate of the overall survival probability of an event of interest. The cumulative incidence is then calculated as one minus this survival probability. Every patient in the data set has a follow-up time and status (event or censored). The follow-up times where an event has occurred are ordered from the smallest to the largest (noting that there can be ties since more than one patient may have the event at the same follow-up time). Consider consecutive event times *t*_*j*−*1*_ and *t*_*j*_. The Kaplan–Meier estimate of the overall survival probability up to event time *t*_*j*_ proceeds as follows. Let *n*_*j*_ be the number of event-free individuals up to time *t*_*j*_. Suppose *d*_*j*_ events have occurred at time *t*_*j*_. The estimated survival probability at time *t*_*j*_ is given by the ratio (*n*_*j*_−*d*_*j*_)/*n*_*j*_. The overall survival probability up to time *t*_*j*_, denoted *S*(*t*_*j*_), is the probability of surviving up to and including time *t*_*j*_. Therefore, the overall survival probability up to *t*_*j*_ is estimated as the product of the probabilities of survival in all the previous times: 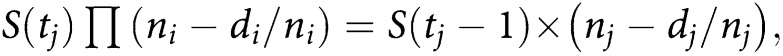 where the product is over *i*=1, …, *j*. The overall survival probability for any time between *t*_*j*−*1*_ and *t*_*j*_ is the same as *S*(*t*_*j*−*1*_), the survival probability up to, but not including, *t*_*j*_.

In the FA data set, it is of interest to estimate the cumulative incidence of HM among FA patients. In order to calculate this, we use the Kaplan–Meier method outlined above to estimate the HM-free survival probability, that is, the probability that an FA patient will not develop HM. We then calculate the cumulative incidence as one minus this survival probability.

Table 1a

**Table 1:** Illustration of the cumulative incidence of haematologic malignancy (HM) for the Fanconi anaemia (FA) data set using the Kaplan–Meier method

(a) A subset of FA patients^a^					
**Patient number**	**Follow-up time**	**Status**	**HM status**		
1	0.003	Dead	C		
2	0.003	Dead	C		
…					
21	4	HM	E		
…					
744	495	HM	E		
745	498	HM	E		
746	509	Alive	C		
747	522	Dead	C		
748	538	Alive	C		
749	544	HM	E		
750	546	HM	E		
751	566	Alive	C		
752	572	Alive	C		
753	582	HM	E		
754	587	Dead	C		
755	599	Alive	C		
					
(b) Kaplan–Meier survival probability estimates^b^					
**Time interval (*t_j_*)**	**# at risk (*n_j_*)**	**# of events (*d_j_*)**	**Survival probability ((*n_j_*−*d_j_*)/*n_j_*) (%)**	**Overall survival (*S*(*t_j_*)) (%)**	**Incidence 1−*S*(*t_j_*)**
0–	755	0	100	100	0
4–	735	1	99.9	99.9	0.1
…				44.9	55.1
495–	12	1	91.7	41.2	58.8
498–	11	1	90.9	37.5	62.5
544–	7	1	85.7	32.1	67.9
546–	6	1	83.3	26.8	73.2
582–	3	1	66.7	17.8	82.2

HM=haematologic malignancy; C=censored; E=event.

a The follow-up time (in months), event status (affected with HM, dead or alive) and HM status (event if patient has HM, censored otherwise).

b Kaplan–Meier survival probability estimates for the subset of the FA patients listed in (a). Column 1 gives the event times (in months). Column 2 shows the number of individuals at risk before that event time. Column 3 is the number of patients diagnosed with HM at that time. Column 4 is the estimated survival probability between that event time and the next. Column 5 provides the overall survival probability using the Kaplan–Meier method. Column 6 gives the cumulative incidence of HM.

illustrates a subset of the Fanconi data set. The individuals were first sorted by their follow-up times (column 2; Table 1a). For example, patient number 21 was diagnosed with HM at the youngest age (4 months). Patients 744 and 745 had HM at 495 and 498 months, respectively, after the onset of FA. Patient 746 was followed for 509 months from birth and was alive at this follow-up time with no diagnosis of HM. Patient 747 died 522 months after the onset of FA without ever being diagnosed with HM. For the Kaplan–Meier analysis, patients 744 and 745 are considered to have had the event (i.e., HM), while patients 746 and 747 are both considered censored at their follow-up times. Patient 747 died prior to the onset of HM, while patient 746 was alive and not diagnosed with HM. However, in the Kaplan–Meier analysis, both these patients will be considered as censored in the same manner.

The estimation of HM-free survival is illustrated in Table 1b. Suppose we are interested in estimating the overall HM-free survival up to time *t*_*j*_=498 months. The previous event occurred at time *t*_*j*−1_=495 months. There were 11 patients with follow-up times of at least 498 months, namely there are 11 patients who lived up to 498 months from the onset of FA and did not develop HM; thus, here *n*_*j*_=11. At 498 months, patient 745 developed HM; thus, *d*_*j*_=1 in this interval. The HM-free survival probability estimate at 498 months is (*n*_*j*_−*d*_*j*_)/*n*_*j*_=(11−1)/11=90.9% (column 4; Table 1b). The overall HM-free survival probability up to 498 months is the probability of surviving without a diagnosis of HM up to 495 months multiplied by the probability of being HM-free at 498 months. The estimated HM-free survival up to 495 months is *S*(*t*_*j*−1_)=41.2%. Hence, the overall HM-free survival probability estimate up to 498 months is *S*(*t*_*j*_)=41.2^*^90.9=37.5% (column 5; Table 1b). The overall HM-free survival probabilities for the other time points can be estimated in a similar manner.

**Figure 1:**
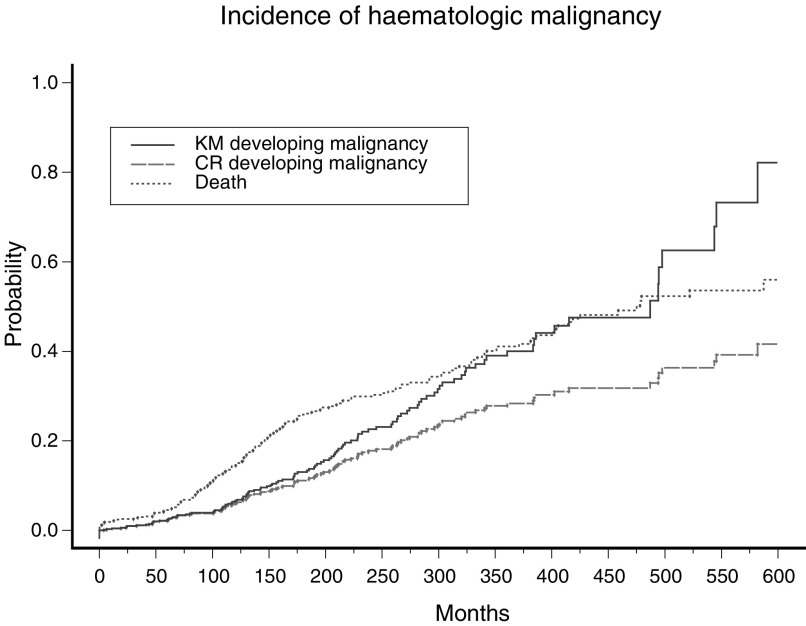
Cumulative incidence of HM in FA patients. The bold line shows the cumulative incidence calculated using the Kaplan–Meier approach without accounting for competing risk events. The dashed line shows the cumulative incidence, after adjusting for competing risk. The dotted line shows the cumulative incidence of the competing risk event (i.e. death occurring prior to the event of interest).

The cumulative disease incidence of developing HM by time *t*_*j*+1_ is one minus the HM-free survival probability, that is, 1−*S*(*t*_*j*+1_). This can be seen in the last column of Table 1b. For example, the HM-free survival or the probability of not developing HM up to 498 months from birth is 41.2%. This is equivalent to a 58.8% probability of developing HM (or cumulative incidence of HM) by this time. Figure 1

provides a graphical representation of the resulting estimates. The estimates derived using the Kaplan–Meier approach are shown using the solid line.

Table 2a

**Table 2:** Illustration of the cumulative incidence of breast cancer-specific mortality for the breast cancer data set using the competing risk approach

(a) A subset of breast cancer patients^a^						
**Patient number**	**Follow-up time**	**Status**	**BC status**			
1	7	Alive	C			
2	10	Dead-BC	E			
3	14	Alive	C			
4	16	Dead-BC	E			
5	18	Dead-Other	CR			
6	23	Dead-BC	E			
7	24	Alive	C			
8	26	Dead-Other	CR			
9	27	Dead-BC	E			
…						
(b) An illustration of estimating cumulative incidence accounting for competing risk for the subset of breast cancer patients listed above (a)						
**Step 1**						
**Time interval (*t_j_*)**	**# at risk (*n_j_*)**	**# of events (*d_j_*)**	**Survival probability ((*n_j_*−*d_j_*)/*n_j_*) (%)**	**Overall survival (*S*(*t_j_*)) (%)**		
0–	305	0	100	100		
10–	304	1	99.7	99.7		
16–	302	1	99.7	99.3		
18–	301	1	99.7	99.0		
23–	300	1	99.7	98.7		
26–	298	1	99.7	98.3		
27–	297	1	99.7	98.0		
…						
						
**Step 2**						
**Time interval (*t_j_*)**	**# at risk (*n_j_*)**	**# of events (*d_j_*_1_)**	**Failure probability (*h*_1_(*t_j_*)) (%)**	**Survival up to time *t_j_* (*S*(*t_j_*_−1_)) (%)**	Incidence (*h*_1_(*t_j_*)*S(*t_j_*_−1_)) (%)	**Cumulative incidence (*I*(*t_j_*)) (%)**
0-	305	0	0	100	0	0
10-	304	1	0.3	100	0.3	0.3
16-	302	1	0.3	99.7	0.3	0.6
23-	300	1	0.3	99.0	0.3	0.9
27-	297	1	0.3	98.3	0.3	1.2
…						

**Figure 2:**
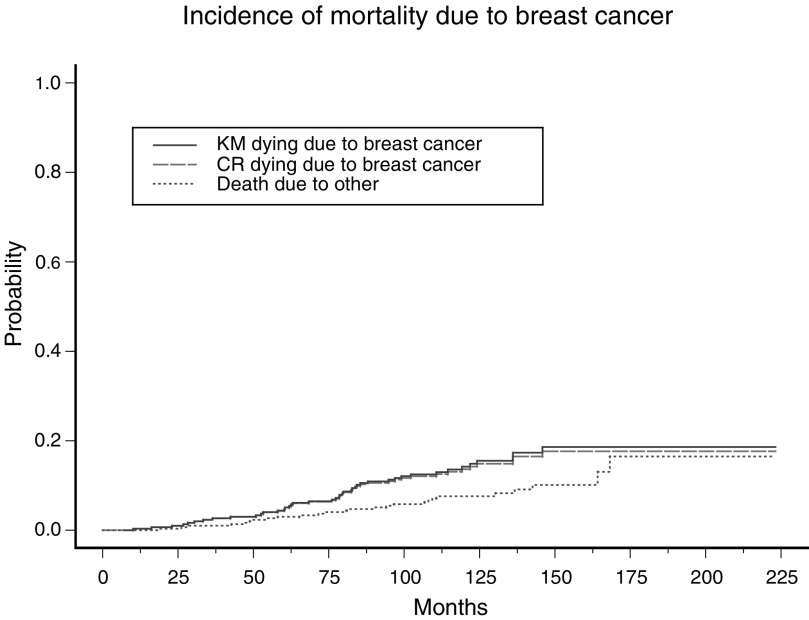
Cumulative breast cancer-specific mortality. The bold line shows the cumulative incidence calculated using the Kaplan–Meier approach without accounting for competing risk events. The dashed line shows the cumulative incidence, after adjusting for competing risk. The dotted line shows the cumulative incidence of the competing risk event (i.e. death due to other causes).

C=censored; E=event; CR=competing risk event.

a The follow-up time (in months), event status (dead due to breast cancer, dead due to causes other than breast cancer, or alive), and breast cancer death status (event if patient died due to breast cancer, censored otherwise).

provides a subset of the breast cancer patients. A graphical representation of the cumulative breast cancer-specific mortality of these patients, estimated using the Kaplan–Meier approach, is given in Figure 2

using a solid line.

### Cumulative incidence estimation in the presence of competing risk events

In this section, we illustrate nonparametric estimation of cumulative incidence of the event of interest taking into account the informative nature of censoring due to competing risks. The cumulative incidence, accounting for competing risk events, is estimated in a two-step process (Kalbfleisch and Prentice, 1980; Marubini and Valsecchi, 1995). In the first step, we calculate the Kaplan–Meier estimate of the overall survival from *any* event. Is it to be noted that here both the event of interest as well as the competing risk event are considered ‘events’. In the second step, the conditional probabilities of experiencing the event of interest are calculated. The cumulative incidences are estimated using these probabilities. The step-by-step calculations are detailed below.

Step 1:

Here we calculate the overall survival probability of being ‘event-free’.

1. An ‘event’ is any event – the onset of the event of interest or the competing risk event. Anyone not experiencing the ‘event’ (i.e. event free) is considered censored.
2. The Kaplan–Meier survival probabilities corresponding to the ‘event’ are calculated as described in the previous section.

Step 2:

Here, we calculate the cumulative probability of experiencing the event of interest.

1. Consider the interval between event-of-interest times *t*_*j*−1_ and *t*_*j*_. (Note that a competing risk event may occur in this interval.)
2. The probability of failure for the *event of interest* is defined as one minus the probability of survival given by *h*(*t*_*j*_)=1−(*n*_*j*_−*d*_*j*_)/*n*_*j*_=*d*_*j*_/*n*_*j*_, where *n*_*j*_ is the number of individuals at risk before time *t*_*j*_ and *d*_*j*_ is the number of *events of interest* occurring at time *t*_*j*_.
3. Now, consider the overall survival probability of surviving any ‘event’ (both the event of interest and the competing risk event) up to, but not including, time *t*_*j*_. This can be obtained from the calculations in Step 1, and is denoted by *S*(*t*_*j*−1_).
4. Accounting for competing risk, the incidence of the event of interest for this interval is estimated as the product *h*(*t*_*j*_)^*^S(*t*_*j*−1_). This can be interpreted as the joint probability of experiencing the event of interest in this time interval given that the individuals survived both the event of interest and the competing risk event in all prior intervals.
5. The cumulative incidence to the end of this time interval is defined as the sum of the incidence in this interval and all previous time intervals.

The cumulative incidence of every distinct type of failure can be calculated as described above. The cumulative incidence of *any* event by a given time will be the sum of the incidence of all distinct failures by that time.

The estimation of the cumulative incidence of breast cancer-specific mortality is outlined in Table 2b. The overall survival from *any* event given under Step 1 of Table 2b is calculated using the Kaplan–Meier approach. In Step 2 of Table 2b, we calculate the probability of the event of interest, that is, death due to breast cancer, in various time intervals. The probability of death due to breast cancer from 10 months up to 16 months is 1/304=0.3%. From Step 1 we know that the probability of surviving (i.e. not dying due to breast cancer or due to any other cause) up to but not including 10 months is 100%. Therefore, the incidence of death due to breast cancer from 10 months up to 16 months is 100% × 0.3%=0.3%. One out of 302 individuals at risk died due to breast cancer at 16 months follow-up time. The next death due to breast cancer occurred at 23 months (patient 6). Therefore, the probability of death due to breast cancer from 16 months up to 23 months is 1/302=0.3%. However, a competing risk event occurred at 18 months (patient 5 died due to other causes). From Step 1, the cumulative probability of surviving any event (i.e. not dying due to breast cancer or due to any other cause) from 18 months up to 23 months is 99%. Therefore, the incidence of the event of interest (death due to breast cancer) from 16 months up to 23 months is 0.3% × 99%=0.3%. Hence, the cumulative incidence of the event of interest up to 23 months is the sum of the incidences in all intervals prior to 23 months, that is 0%+0.3%+0.3%=0.6%.

Figure 2 illustrates the cumulative incidence corresponding to death due to breast cancer. The long dashed line is the cumulative incidence of the event of interest, that is, death due to breast cancer. The dotted line is the cumulative incidence of the competing risk event, that is, death due to other causes. Similarly, the cumulative incidence of HM and cumulative mortality prior to the onset of HM for the FA data set are shown in Figure 1 using long dashed and dotted lines, respectively.

It is to be noted that the cumulative incidence of *any* event is the sum of the cumulative incidence of the event of interest and the cumulative incidence of the competing risk events. Therefore, the cumulative mortality among the breast cancer patients is the sum of the cumulative breast cancer-specific mortality and the cumulative mortality due to causes other than breast cancer. Similarly, in the FA data set, the cumulative incidence of experiencing any event (i.e. developing HM or death prior to the onset of HM) is simply the sum of cumulative incidence of HM and the cumulative mortality without the onset of HM.

### Cumulative incidence of multiple groups

Often it is of interest to estimate (and compare) the cumulative incidences between two or more groups. For example, in the FA data set, it may be of interest to estimate the incidence of HM in the various complementation groups. Likewise, in the breast cancer data, it may be of interest to estimate breast cancer-specific mortality for those with and without a BRCA mutation. This is carried out by first dividing the sample into the subgroups of interest. The cumulative incidences of the event of interest are then calculated for each group separately as outlined above. Table 3

**Table 3:** Cumulative incidence of haematologic malignancy in Fanconi anaemia patients obtained using the Kaplan–Meier (KM) approach by not adjusting for competing risk events, and estimated by adjusting for competing risk events (CR)

		**Overall (%)**	**A (%)**	**C (%)**	**G (%)**	**O (%)**
	**N (HEM)**	**755 (120)**	**207 (30)**	**78 (19)**	**46 (10)**	**414 (60)**
% Censored	KM	84	86	76	78	86
	CR	58	67	44	57	55
10 year	KM	6.3	5	15	11	5
	CR	5.9	4	11	7	5
20 year	KM	22.6	20	42	32	19
	CR	17.8	15	27	21	14
30 year	KM	39.0	45	53	45	34
	CR	27.8	31	32	31	23
40 year	KM	47.8	52	68	45	44
	CR	31.8	34	35	31	27

Column 3 (‘Overall’) shows the cumulative incidence for all the 755 patients. Columns 4, 5, 6 and 7 show the cumulative incidence estimates for patients in complementation groups A, C, G and O (mostly nontyped patients and a small number of patients in uncommon complementation groups). The sample size is denoted N. The number of haematologic malignancy events is denoted by HEM and is given in parentheses in the second row.

provides the cumulative incidences of HM using the Kaplan–Meier approach as well as the competing risks approach, separately for patients in complementation groups FA-A, -C, -G and other patients (O=mostly nontyped patients and a small number of patients in uncommon complementation groups). Likewise, Table 4

**Table 4:** Breast cancer-specific mortality obtained using the Kaplan–Meier (KM) approach by not adjusting for competing risk events, and estimated by adjusting for competing risk events (CR)

		**Mortality due to breast cancer**		
		**Overall (%)**	**BRCA mutation (%)**	**No mutation (%)**
	**N (BCSS)**	**305 (43)**	**28 (8)**	**276 (35)**
% Censored	KM	86	71	87
	CR	77	64	79
1 year	KM	0.3	3.6	0.0
	CR	0.3	3.6	0.0
5 year	KM	4.4	10.9	3.7
	CR	4.3	10.7	3.7
10 year	KM	14.2	28.4	12.8
	CR	13.6	27.0	12.3
15 year	KM	18.6	37.3	16.7
	CR	17.6	35.2	15.9

Column 3 (‘Overall’) shows the cumulative incidence for all the 305 breast cancer patients. Columns 4 and 5 show the cumulative incidence estimates for patients with and without a BRCA mutation. The sample size is denoted N. The number of breast cancer-specific deaths is denoted BCSS and is given in parentheses in the second row. One patient without a BRCA mutation had missing death status and hence was excluded from the analysis.

provides breast cancer-specific mortality for patients with and without a BRCA mutation using the two methods.

The cumulative incidences in the various groups can be compared using nonparametric tests, namely the log-rank test (Kalbfleisch and Prentice 1980) when calculating incidences based on the Kaplan–Meier approach or a modified *χ*^2^ test (Gray, 1988) when calculating incidences in the presence of competing risks. The cumulative incidence estimation methods outlined above are nonparametric, that is, these estimates are not based upon any specific model. Alternative model-based approaches can also be utilized to estimate cumulative incidences of specific events, adjusting for prognostic factors of interest. Under the assumption of noninformative censoring, the Cox proportional hazards model (Cox, 1972) can be used. In the presence of competing risk events, a modified Cox proportional hazards model or the competing risk regression approach has been developed by Fine and Gray (1999). We do not detail these methods here, but refer the reader to the references provided above.

## DISCUSSION

In this paper, we have focused on the estimation of cumulative incidence function for an event of interest in the presence of competing risk events. We have outlined nonparametric estimation using a Kaplan–Meier approach, which assumes noninformative censoring, as well as an alternative approach that accounts for informative censoring. Calculating the standard error of the estimates and the corresponding confidence intervals are discussed by Collett (1994) and Marubini and Valsecchi (1995). The Kaplan–Meier approach results in one curve that portrays the estimated cumulative probability of the event of interest (i.e. one minus the estimated survival probability) with a jump in the curve corresponding to the occurrence of an event of interest at a specific time. The competing risk approach generates two curves, one representing the event of interest and the other representing the competing risk event. The curve representing the cumulative incidence of the event of interest has jumps at times where an event of interest occurs. Likewise, the curve corresponding to the incidence of the competing risk event has jumps at times where competing risk events occur.

The estimated cumulative incidence of an event of interest derived using the Kaplan–Meier approach is, in general, larger than estimates obtained when accounting for competing risks. This is due to the following reason. In the Kaplan–Meier estimation approach, when an individual experiences a competing risk event, this individual is treated as censored and is eliminated from the risk set. On the other hand, in the competing risk approach, this individual is an event in the calculation of the overall survival probability of *any* event (Step 1). The estimated overall survival of any event is lowered when this individual experiences a competing risk event. Recall that the incidence of the event of interest in a specific time interval is the probability of surviving any event up to that time interval and experiencing the event of interest in that interval. Since the overall survival is reduced when any event occurs, the resulting incidence of the event of interest is reduced as well.

In the FA data set, a total of 120 out of the 755 patients were diagnosed with HM. The remaining 635 patients not diagnosed with HM were considered censored under the Kaplan–Meier approach. The estimated cumulative incidences using the Kaplan–Meier approach are 6.3, 22.6, 39.0 and 47.8% at 10, 20, 30 and 40 years, respectively, since birth (Table 3). A total of 199 out of the 635 ‘censored’ patients died prior to the onset of HM. The estimated cumulative incidences of HM using the competing risk approach are 5.9, 17.8, 27.8 and 31.8%, respectively, at 10, 20, 30 and 40 years since the diagnosis of FA. Thus, the estimates are lower when accounting for the competing risk event. Table 4 summarises the cumulative incidence estimates for the breast cancer data set. A total of 43 out of the 305 patients died due to breast cancer. The remaining 262 individuals were considered censored when estimating the cumulative mortality without accounting for competing risk using the Kaplan–Meier approach. Of these 262 individuals, 25 women died due to causes unrelated to breast cancer. The estimated cumulative breast cancer-specific mortality without accounting for competing risk are 0.3, 4.4, 14.2 and 18.6% at 1, 5, 10 and 15 years, respectively. The corresponding estimates when accounting for competing risks are 0.3, 4.3, 13.6 and 17.6%, respectively. As before, it is evident that the estimates are lower when accounting for the competing risk event.

In certain situations, the cumulative incidence of an event of interest estimated using the Kaplan–Meier approach and the competing risk approach can be similar. The difference between the estimated breast cancer specific-mortality derived from the Kaplan–Meier and the competing risk approach is small for the breast cancer data set. However, the incidence of HM estimated using the Kaplan–Meier approach is substantially larger than that derived from the competing risk approach. Fanconi anaemia patients are at increased risk for HM as well death, relative to the general population (Kutler *et al*, 2003), due to their underlying disease, that is, FA. It is, therefore, important in this setting to appropriately account for the competing causes of risk when estimating the cumulative incidence of HM. On the other hand, death due to other causes may not be related to having breast cancer unlike breast cancer-specific mortality. In this case, ignoring the informative censoring mechanism does not substantially influence the estimates of breast cancer-specific mortality.

When there are no competing risk events, that is, when there is only one type of failure, the estimate of the cumulative incidence of the event derived using the Kaplan–Meier approach and the competing risk approach will be identical. Similarly, in the setting when it is of interest to estimate the cumulative incidence of the first event in the presence of multiple types of failure, there are no competing risk events. This is because any event that occurs subsequent to the first event is not relevant to the analysis. Only those patients not experiencing any event will be censored. For example, suppose an FA patient dies after experiencing HM, and it is of interest to estimate the cumulative incidence of the first event (HM or death). In this setting, only the time to HM would be of interest for this patient in the cumulative incidence calculation. The estimated cumulative incidence of the first event obtained using the Kaplan–Meier approach will be the same as that obtained using the competing risk approach.

One minus the Kaplan–Meier survival probability can be interpreted as the cumulative probability of failure. The cumulative incidence of an event of interest estimated by accounting for competing risk events is the probability of experiencing the event of interest by a given time and not experiencing a competing risk event by this time. One minus the cumulative incidence is the probability of surviving the event of interest up to a specific time. This can occur if the patient did not experience both the event of interest and the competing risk event, or if the patient had the competing risk event before the onset of the event of interest. As a result, one minus the cumulative incidence adjusted for competing risk events cannot be interpreted as the probability of surviving *any* event.

The topics of competing risk events and the estimation of cumulative incidence of an event of interest have been discussed by several authors. Gail (1975) reviews the theoretical concepts underlying the estimation of cumulative incidence of an event using a variety of models. Prentice *et al* (1978) discuss likelihood inference to examine the effect of prognostic factors on the event of interest in the presence of competing risk events. Pepe and Mori (1993) describe various probability models for summarising competing risk data. Tai *et al* (2001) developed a method to estimate the cumulative incidence of a specific event based on an extension of the Cox proportional hazards regression model. They compare their estimates to the Kaplan–Meier estimate of cumulative incidence as well as the cumulative incidence accounting for competing risk as described above. Their findings show that the estimates obtained using the Kaplan–Meier approach are numerically larger than those accounting for competing risk events. Clark *et al* (2003a, 2003b) and Bradburn *et al* (2003a, 2003b) provide a detailed tutorial review of various survival analysis concepts, including a brief summary of competing risk analysis.

Several softwares are available to estimate the overall survival probabilities and cumulative incidence of an event of interest. The Kaplan–Meier estimate of survival probability can be easily obtained using standard statistical analysis softwares including R (http://www.r-project.org), S-plus (Insightful Corp., 2003), SAS (SAS Institute, 2002) and SPSS (SPSS Inc., 2003). Software has been made available in R by Gray (1988; http://biowww.dfci.harvard.edu /~gray/) for obtaining the estimate of cumulative incidence in the presence of competing risk events.

In summary, it is important to account for competing risk events when estimating disease incidence. Failure to account for such competing events results in an overestimate of the cumulative incidences. This could be substantial when the competing risk event is related to or is a result of the underlying disease.
